# Evaluating burnout among clinical pharmacists across 23 governorates in Egypt: a cross-sectional study

**DOI:** 10.1186/s40359-025-03669-x

**Published:** 2025-12-06

**Authors:** Mohamed Hendawy, Mohamed Abouzid, Heba Elshazly, Ali Ahmed Ali Ismeal, Mahmoud Elazb, Khaled Moghib, Omnia Hemdan, Marwa Elnagar, Ghadeer Awad

**Affiliations:** 1 Faculty of Pharmacy, Alexandria University, Alexandria, Egypt; 2 Department of Physical Pharmacy and Pharmacokinetics, Faculty of Pharmacy, Poznan University of Medical Sciences, Rokietnicka 3 St, 60-806 Poznan, Poland; 3 Doctoral School, Poznan University of Medical Sciences, 60-812 Poznan, Poland; 4 Faculty of Pharmacy, Assiut University, Assiut, Egypt; 5 Pharm D Division, Suez Canal University, Ismailia, Egypt; 6 Faculty of Pharmacy, Minia University, Minia, Egypt; 7 Faculty of Pharmacy, Al-Azhar University, Cairo, Egypt; 8 Faculty of Medicine, Kasr Al-Ainy Cairo University, Cairo, Egypt; 9 Medical research group of Egypt, Negida Academy, Arlington, MA, United States; 10 Pharmaceutical Toxicology Department, Faculty of Pharmacy, Hacettepe University, Ankara, Turkey; 11 Faculty of Pharmacy, MTI University, Cairo, Egypt

**Keywords:** Burnout, Copenhagen Burnout Inventory, Clinical pharmacists

## Abstract

**Background:**

The growth in services provided by clinical pharmacists in Egypt and their collaborative work with physicians contribute to enhancing patient safety and realizing favorable clinical outcomes. Several studies highlighted that clinical pharmacists may be susceptible to burnout. Hence, we are conducting this study due to a lack of research evaluating the prevalence of burnout among clinical pharmacists in Egypt.

**Methods:**

We conducted a cross-sectional study on clinical pharmacists in Egypt from October 2022 to January 2023. The questionnaire included demographic characteristics and Copenhagen Burnout Inventory (CBI). A *p*-value less than 0.05 was considered statistically significant in all tests.

**Results:**

Two hundred twenty-two clinical pharmacists filled out the survey, 75% had moderate and above burnout levels (CBI ≥ 50). Three multivariate models were built to explore burnout across personal, work-related, and patient-related domains. Our key findings indicate that individuals facing financial strain (insufficient income and debt) are significantly more likely to experience higher burnout. Additionally, those with a master's degree (compared to a bachelor's in pharmacy) are more prone to work-related burnout. Furthermore, an association exists between more years of experience and elevated levels of both work and personal burnout. Lastly, direct patient contact and being female are linked to higher personal burnout.

In terms of univariate predictors for high overall burnout (CBI ≥ 75), more years of experience and being in debt are significantly associated with higher odds of burnout (with odds ratios of 1.08 and 6.00, respectively). The back stepwise regression model reinforces the impact of years of experience, suggesting that for each additional year of experience, the odds of high burnout increase by approximately 8.4%

**Conclusion:**

The prevalence of burnout among clinical pharmacists is high. Demographic characteristics may significantly influence burnout levels within specific domains. Notably, pharmacists with more years of experience are more likely to experience high burnout. These findings are alarming and warrant further investigation. Proper interventions are needed to identify the reasons behind this trend among more experienced pharmacists. By identifying these factors, we can develop effective strategies to mitigate burnout and promote well-being in this group of professional healthcare providers.

**Graphical Abstract:**

Information was collected from 222 clinical pharmacists across 32 governorates in Egypt. The names of the governorates are displayed on the left side, with each color representing a different governorate. The frequency of responses from each governorate is indicated by columns on the map; the taller the column, the higher the frequency from that governorate. Demographic data is also represented, including gender (female and male), residency (rural or urban), highest education level (Doctorate, PhD; Master, MSc; or Bachelor, BSc), and income status($, Not enough and in debt; $$, Not enough; $$$, Just enough; or $$$$, Enough and can save). We also represented the average age (in years), experience (in years), and Copenhagen Burnout Inventory average scores for each domain for all responders.

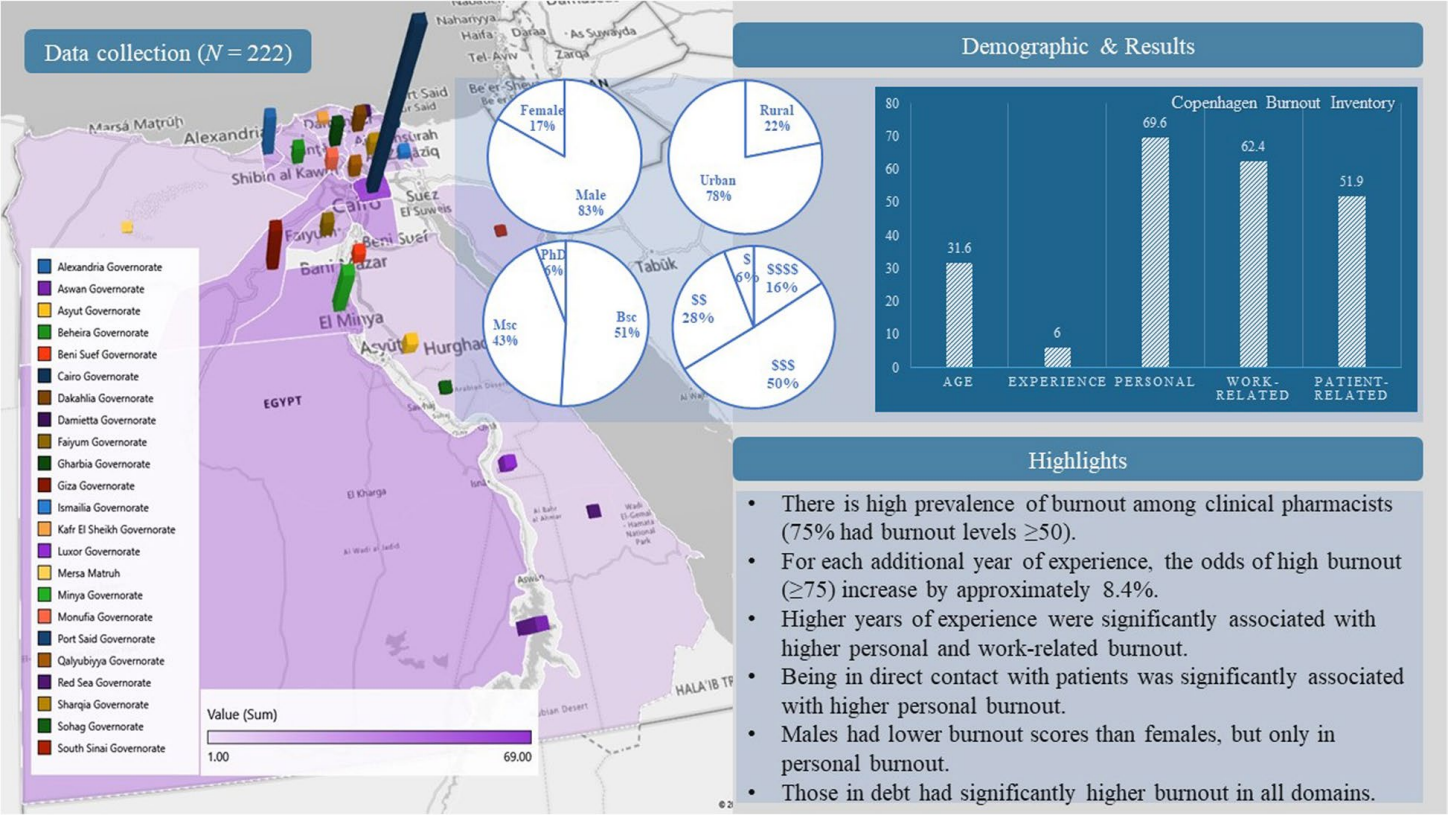

## Introduction

Occupational burnout is a well-recognised threat to healthcare quality and workforce sustainability. Maslach and Leiter frame it as emotional exhaustion, cynicism, and reduced professional efficacy that arise from prolonged work stress [1]. Five decades of scholarship implicate excessive workload, effort-reward imbalance, poor autonomy, and mismatched organisational values as common antecedents [1, 2]. Although burnout is not a formal psychiatric diagnosis, its physical and psychological sequelae are wide-ranging – chronic fatigue, somatic complaints, anxiety, and depression – and translate into absenteeism, turnover, malpractice, and diminished patient satisfaction [3, 4].

Collaboration between clinical pharmacists and other providers has reduced workload, improved medication use, lowered errors, and supported patient health goals [5]. Integration also boosts satisfaction – 91% of providers in one study were delighted with clinical pharmacy services [6]. Yet burnout is a concern: a U.S. study found a 61.2% burnout rate among hospital clinical pharmacists (11.4% response rate) [7], and in Vietnam, 52.8% of 197 clinical pharmacists (82.4% response rate) reported job stress [8].

In the Middle East, studies from Saudi Arabia, Qatar, Lebanon, Egypt, and the UAE highlight burnout's widespread prevalence and causes among healthcare providers, particularly pharmacy professionals. In Saudi Arabia, a study conducted during the COVID-19 pandemic linked burnout to long hours, heavy workloads, and limited control over work, particularly affecting younger pharmacists [9]. In Qatar, tension, lack of teamwork, and recognition were key burnout factors, although rates were lower than international levels [10]. Lebanon reported high burnout rates among community pharmacists [11], while Egypt found significant burnout among healthcare providers [12]. The UAE study revealed high burnout and depression rates among medical residents [13], emphasizing the need for interventions.

In Egypt, pharmacy practice is regulated by the Egyptian Pharmacists Syndicate, with distinctions between general pharmacists and clinical pharmacists. Clinical pharmacists, who focus on direct patient care and medication therapy management, require additional training beyond the standard pharmacy degree. While both general and clinical pharmacists must be licensed, clinical pharmacists often undergo specialized postgraduate education or certification to practice in hospitals and healthcare settings. The expansion of services by clinical pharmacists in Egypt, combined with their collaborative efforts with physicians, plays a crucial role in improving patient safety and achieving positive clinical outcomes [14].

However, there is a notable lack of studies assessing the average prevalence and severity of burnout among clinical pharmacists in Egypt, making it unclear how common the issue is in this context. Furthermore, although international and regional studies have linked burnout to personal and professional characteristics, these relationships remain unexplored among Egyptian clinical pharmacists. Potential predictors relevant to this setting include age, gender, residency, education level, working hours, years of experience, family income, dependent children, direct patient contact, and presence of chronic disease. We defined direct patient contact as face-to-face clinical interaction with patients or caregivers (e.g., medication history/reconciliation, bedside rounds, clinics, counseling/education, or discharge counseling).

The principal objective of this study is to evaluate burnout among clinical pharmacists working in inpatient hospital settings in Egypt. Specifically, it aims to answer:

1. What is the average level of burnout among clinical pharmacists?
2. How do demographic factors (e.g., gender, age, residency, education level, income status, and direct patient contact) correlate with burnout levels?
3. What factors predict higher burnout levels?

## Methods

### Study design

We performed a cross-sectional study following the Strengthening the Reporting of Observational Studies in Epidemiology (STROBE) guidelines [15]. We used an anonymous, self-administered online survey tool through the "Google Forms" platform.

### Study population

Clinical pharmacists practicing in Egypt were eligible. Inclusion criteria were: (i) full-time employment in a clinical pharmacist role that provides direct patient care in a hospital or health-system setting; (ii) minimum qualification of B.Pharm (higher degrees such as PharmD/MSc/PhD acceptable); (iii) active pharmacist license issued by the Egyptian Pharmacists Syndicate; (iv) ≥ 1 year of experience in the clinical pharmacist role; and (v) age ≥ 18 years.

License status and role were self-reported via screening items (job title, practice setting, license status); no registry lookup or license-number verification was performed.

Exclusion criteria were students, pharmacy interns/pre-registration pharmacists, part-time or non-clinical roles, and surveys with substantial missing or internally inconsistent data. No restrictions were applied by gender, governorate, educational level, or socioeconomic status.

### Sampling

The questionnaire, including the Copenhagen Burnout Inventory (CBI), was administered in English. Given that pharmacy education and routine professional communication in Egypt are delivered in English, no translation or cultural adaptation of the instrument was required. The CBI is open-access and does not require a paid licence or formal permission for research use; thus, none was sought.

We initially distributed the questionnaire to clinical pharmacist colleagues who met the inclusion criteria from October 2022 to January 2023. We then asked them to share it with their colleagues -clinical pharmacists- to create a snowball effect and maintain the inclusion criteria. Additionally, to reach the target audience further, we shared the survey through professional and social media channels such as LinkedIn, Facebook, and WhatsApp. We clearly stated that the survey was intended only for clinical pharmacists who met the specific inclusion criteria. To verify eligibility, the survey included screening questions related to these criteria (e.g., years of experience, working hours per week), ensuring that only qualified participants could proceed – no license or qualifications have been verified to keep the anonymous desin of the study.

Notably, we aimed to include all Egyptian 27 governorates (Alexandria, Aswan, Asyut, Beheira, Beni Suef, Cairo, Dakahlia, Damietta, Faiyum, Gharbia, Giza, Ismailia, Kafr El Sheikh, Luxor, Matrouh, Minya, Monufia, New Valley, North Sinai, Port Said, Qalyubia, Qena, Red Sea, Sharqia, Sohag, South Sinai, Suez).

OpenEpi was used to calculate the sample size. Assuming a prevalence rate of burnout of 17.6% (*P* = 0.176), similar to that reported by Alharbi et al. [16], with a 95% confidence level (*Z* = 1.96) and 5% margin error (*E* = 0.05), it was be required to have approximately 223 clinical pharmacists to detect a similar prevalence rate, calculated as below:

$$A=\frac{{Z}^{2}\times P\times (1-P)}{{E}^{2}}$$

### Study tool

We divided the survey into two sections:

1. The demographic characteristics (age, gender, pharmacist working hours per week, dependent children, years of experience, residency, highest education level, direct contact with the patient, and household economic status (income sufficiency)). We measured perceived household economic status using a single-item, four-category, self-report of income sufficiency adapted from commonly used public-opinion and living-conditions surveys. Participants selected one option that best described their household situation: (1) “Enough and save,” (2) “Just enough,” (3) “Not enough,” (4) “Not enough and in debts.” These categories align with the Arab Barometer income sufficiency item (“covers and can save” → “significant difficulties”), and with the EU-SILC “ability to make ends meet” framework; we retained locally intuitive wording for clarity [17, 18].
2. We selected the CBI as our primary burnout measure [19]. The CBI is a validated [20], non-commercial instrument [20] that assesses personal, work-related, and client/patient-related burnout, capturing both general exhaustion and patient-facing demands relevant to clinical pharmacy practice. Importantly, the CBI has been validated in pharmacists [21] and adapted/validated in Arabic among community pharmacists, with growing use in healthcare research in low- and middle-income settings [22, 23], which supports its suitability for Egypt. In contrast, the widely used Maslach Burnout Inventory (MBI) is a proprietary instrument requiring paid licensing [24].

CBI consists of 19 questions that cover three aspects: personal burnout (six items), work-related burnout (seven items), and patient-related burnout (six items) [19]. The survey answers were scored from 0, 25, 50, 75, and 100 points. Following widely used thresholds in CBI studies, we categorized 0–49 = low, 50–74 = moderate, 75–99 = high, and 100 = severe burnout [25, 26]. More information on questions and internal consistency reliability is reported in the results section.

Notably, we operationalized “direct patient contact” as in-person clinical encounters with patients or caregivers as part of the pharmacist’s role (e.g., obtaining medication histories, inpatient ward/bedside rounds, ambulatory clinics, counseling/education, device training, adverse-event follow-up, or discharge counseling). Roles without patient-facing time – such as sterile compounding/IV admixture, central distribution/dispensing without counseling, verification-only, procurement, or quality assurance – were coded as no direct patient contact. The item was captured as a binary variable (Yes/No).

### Ethical consideration

We conducted this study according to the Declaration of Helsinki [27]. Written informed consent was obtained electronically from all participants – the consent was implied by selecting an agreement option within the Google Form. Participation in the online survey was entirely voluntary; participants could exit the survey at any time without penalty. Additionally, all responses were reported and analyzed anonymously to ensure participants' confidentiality throughout the research process. The study received ethical approval from the faculty of Pharmacy, Minia University's ethical approval committee (MPEC 230109).

### Statistical analysis

We performed the statistical analysis using PQStat v.1.8.2.238. Pairwise deletion was used to handle missing data. The Shapiro–Wilk test was employed to assess the normality of continuous data. Categorical data were reported as frequency/percentage, and continuous data as mean/standard deviation (SD) for normal distribution or median/interquartile range (IQR) for non-normal distribution. The Mann–Whitney U test was used to measure the differences in personal, work-related, and patient-related burnout across gender, residency, presence of chronic disease, and direct patient contact. The Kruskal–Wallis ANOVA with post-hoc (Dunn Bonferroni) was used to test the differences in personal, work-related, and client-related burnout (client, in this case, can be referred to as patient) among pharmacists' highest level of education and economic status. Spearman's rank correlation coefficient (r) was used to test the correlation between personal, work-related, and patient-related burnout and continuous data (age, working hours per week, and years of experience). We built a multi-logistic regression model for each burnout domain. Finally, we constructed univariate and back-stepwise multi-logistic regression models to predict pharmacists with an overall high burnout level (CBI ≥ 75 [26]). The results were presented as odds ratios (OR) and 95% confidence intervals (CI). A p-value less than 0.05 was considered statistically significant in all tests.

## Results

### Demographic characteristics of the participants

Two hundred twenty-two clinical pharmacists completed the survey from 23 governorates in Egypt (Alexandria, Aswan, Asyut, Beheira, Beni Suef, Cairo, Dakahlia, Damietta, Faiyum, Gharbia, Giza, Ismailia, Kafr El Sheikh, Luxor, Mersa Matruh, Minya, Monufia, Port Said, Qalyubiyya, Red Sea, Sharqia, Sohag, and South Sinai). The average age of the participants was approximately 31.6 years, and they worked an average of 40.3 h per week. On average, pharmacists reported having one dependent child. Their professional experience spanned approximately six years. Regarding burnout, the participants experienced varying levels: personal burnout averaged 69.6, work-related burnout stood at 62.4, and patient-related burnout was around 51.8 **(**Table 1).

**Table 1:** Demographic characteristics of clinical pharmacists who participated in the survey (*N* = 222), reported as n (%)

Sociodemographic, economic, and work-related data	
Age	31.6 ± 5.4; [30 (27–35)]^‡^
Gender	
Female	38 (17.1)
Male	184 (83.9)
Residency	
Rural	49 (22.1)
Urban	173 (77.9)
Highest education level	
Bachelor of Pharmacy (B.Pharm)	113 (50.9)
Master (M.Sc.)	95 (42.8)
Doctor of Philosophy (Ph.D.)	14 (6.3)
Direct contact with the patient	
No	22 (9.9)
Yes	200 (90.1)
Family income/economic level	
Enough and save	35 (15.8)
Just enough	112 (50.5)
Not enough	61 (27.5)
Not enough and in debt	14 (6.3)
Pharmacist working hours per week	40.3 ± 15.0; [36 (36–48)]^‡^
Dependent children	1 ± 1.2; [0 (0–2)]^†‡^
Years of experience	6.0 ± 4.8; [5 (2–10)]^‡^
Burnout results	
Personal burnout	69.6 ± 17.3; [66.7 (54.2–83.3)]^‡^
Work-related burnout	62.4 ± 14.9; [60.7 (50.9–71.4)]^‡^
Patient-related burnout	51.8 ± 20.2; [50 (37.5–66.7)]^‡^
Overall burnout score	61.2 ± 14.7; [60 (50.1–72.3)]^‡^
Low (< 50)	54 (24.3)
Moderate (50 to 74)	122 (55.0)
High (75–99)	45 (20.3)
Severe (100)	1 (0.5)

^†^missing three data points

^‡^reported as mean ± SD; median (IQR)

Additionally, the study explored other factors such as gender distribution, residency (rural vs. urban), education levels, direct patient contact, and family income. Notably, 83.9% of the participants were male, and 77.9% resided in urban areas. Most held a bachelor's degree in pharmacy (B.Pharm) (50.9%), while others had completed a master's program (M.Sc.) or doctor of philosophy (Ph.D.). Most had regular patient interactions (90.1%), and their economic situations varied from sufficient income and savings to financial challenges and debt (Table 1).

### Frequencies of responding and validation

The scales' internal consistency reliability was determined with Cronbach's standardized alpha and demonstrated good reliability of Personal Burnout (α 0.87), Work-related Burnout (α 0.72) and Patient-related Burnout (α 0.82) [28]. The frequency of response to each question is shown in Table 2.

**Table 2:** Copenhagen Burnout Inventory (CBI). Scales, items, and response frequencies

	Response category and scoring					
	Always Or To a very high degree	Often or To a high degree	Sometimes or Somewhat	Seldom or To a low degree	Never/almost never or To a very low degree	Score Mean (SD)
	(Scoring 100)%	(Scoring 75)%	(Scoring 50)%	(Scoring 25)%	(Scoring 0)%	
***Personal burnout*** (α 0.87) (***N*** = 222)						
How Often do you feel tired?^a^	33.8	34.2	31.1	0.9	0	75.3 (20.8)
How Often are you physically exhausted?^a^	29.7	39.2	28.8	1.8	0.5	74 (21)
How Often are you emotionally exhausted?^a^	34.7	34.7	27.9	2.3	0.5	75.3 (21.8)
How Often do you think: "I can't take it anymore"?^a^	22.5	30.6	36.9	8.1	1.8	66 (24.5)
How Often do you feel worn out?^a^	20.7	35.6	38.3	5.0	0.5	67.8 (21.7)
How Often do you feel weak and susceptible to illness?^a^	12.2	26.6	47.3	13.5	0.5	59.2 (22.1)
Total average score						69.6 (17.3)
Work-related burnout (α 0.72) (N = 222)						
Is your work emotionally exhausting?^b^	23	36	31.1	8.6	1.4	67.7 (24.1)
Do you feel burnt out because of your work?^b^	26.1	37	31.1	5.4	0.5	70.8 (22.5)
Does your work frustrate you?^b^	14.4	28.8	40.1	12.2	4.5	59.2 (25.5)
Do you feel worn out at the end of the working day?^a^	25.2	37.9	30.2	5.4	1.4	70.1 (23.2)
Are you exhausted in the morning at the thought of another day at work?^a^	29.8	26.1	32.8	10	1.4	68.3 (26)
Do you feel that every working hour is tiring for you?^a^	11.7	24.8	46	13.5	4.1	56.7 (24.4)
Do you have enough energy for family and friends during leisure^a^	3.6	16.2	41.4	29.3	9.5	43.9 (24)
Total average score						62.4 (14.9)
*Client-related burnout* (α 0.82) (*N* = 222)						
Do you find it hard to work with patients?^b^	8.1	15.3	39.6	26.6	10.4	46.1 (26.7)
Does your work frustrate you?^b^	14.4	28.8	40.1	12.2	4.5	59.2 (25.5)
Does it drain your energy to work with patients?^b^	10.4	22.5	34.2	19.4	13.5	49.3 (29.4)
Do you feel that you give more than you get back when you work with patients?^b^	20.3	23.4	29.3	18	9	57 (30.8)
Are you tired of working with patients?^a^	4.1	16.2	32.4	30.6	16.7	40.1 (26.8)
Do you wonder how long you will be able to continue working with patients?^a^	20.7	22.5	38.3	10.8	7.7	59.5 (28.9)
Total average score						51.9 (20.3)

Possible score range for all scales is 0 to 100

a. Response categories for items denoted with^a^

b. Response categories for items denoted with^b^

### Demographic characteristics and levels of burnout

Burnout levels were generally high across domains. Mean (± SD) scores were 69.6 ± 17.3 for personal burnout, 62.4 ± 14.9 for work-related burnout, and 51.8 ± 20.2 for patient-related burnout, with corresponding medians [IQR] of 66.7 [54.2–83.3], 60.7 [50.9–71.4], and 50.0 [37.5–66.7], respectively. Emotional exhaustion (personal burnout) was the most pronounced dimension, followed by work-related strain. Patient-related burnout was comparatively lower and closer to the mid-scale. The sizeable SDs/IQRs indicate notable variability among pharmacists, especially for the personal and patient-related subscales.

Overall burnout was concentrated in the moderate range but with a high-burnout tail. The overall CBI mean was 61.2 ± 14.7 (median 60.0 [50.1–72.3]). Categorically, 24.3% fell in the low range (< 50), 55.0% in moderate (50–74), 20.3% in high (75–99), and 0.5% in severe (100). Thus, ~ 76% of pharmacists reported at least moderate burnout, and ~ 21% met high-or-severe criteria.

**Fig. 1:**
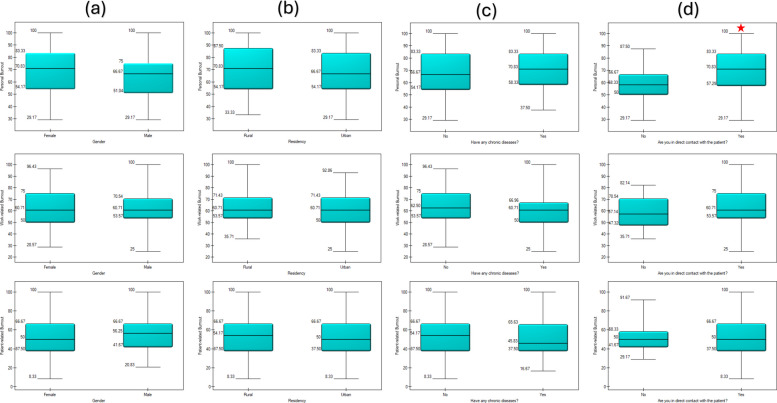
Mann–Whitney U test for differences in personal, work, and patient-related burnout according to (**a**) gender; (**b**) residency; (**c**) having chronic disease; and (**d**) being in direct contact with patients. Red stars denote statistically significant differences

**Fig. 2:**
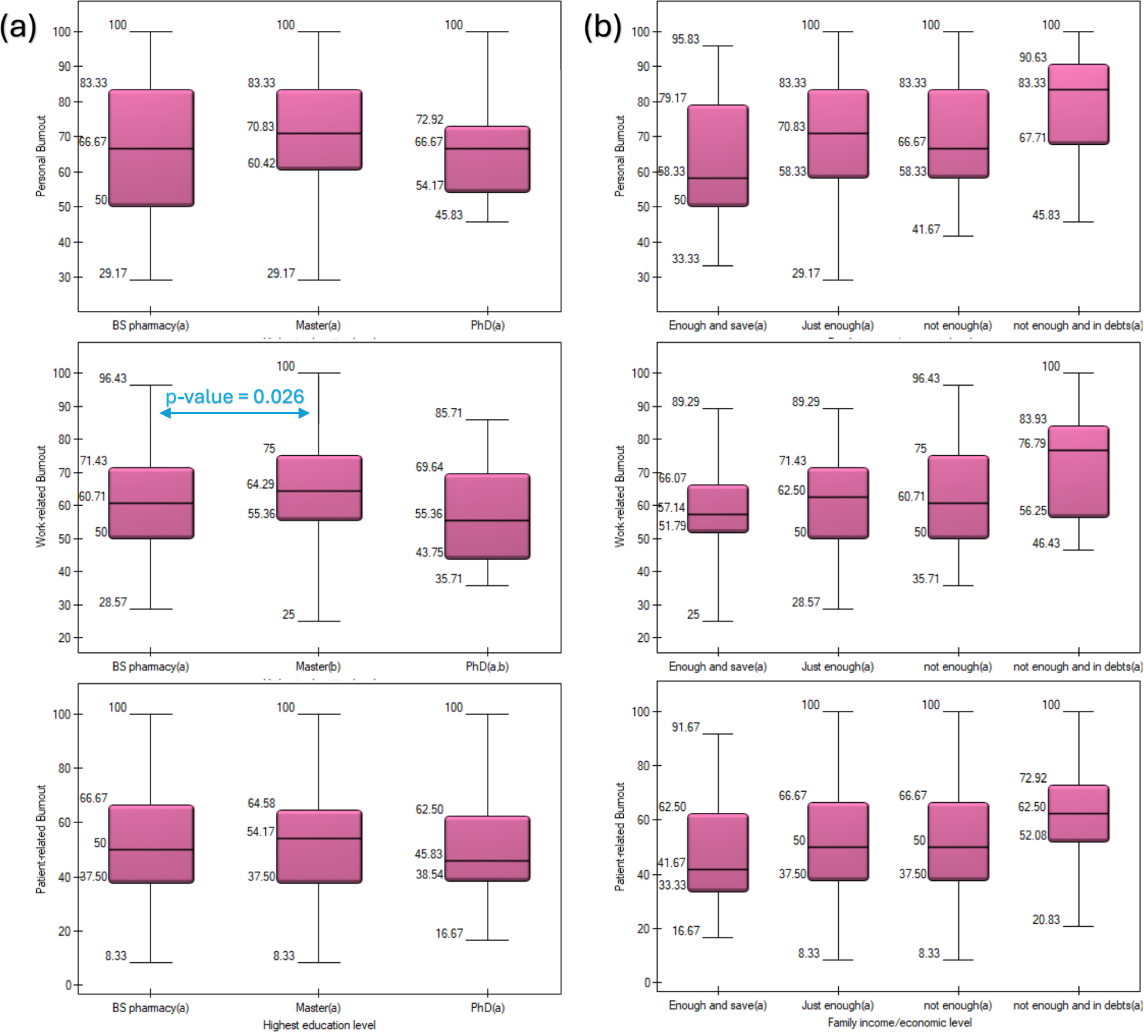
Post-hoc (Dunn-Bonferroni) analysis to test the differences in personal, work, and patient-related burnout among clinical pharmacists according to (**a**) highest education and (**b**) economic status. Significant differences between variables are marked with blue *p*-values

Subgroup analysis showed that pharmacists with direct patient contact had significantly higher personal burnout than those without [70.8 (57.3–83.3) vs. 58.3 (50–66.7), *p* = 0.006]. Pharmacists with M.Sc. degrees reported higher work-related burnout than those with B.Pharm [64.3 (55.4–75) vs. 60.7 (50–71.4), *p* = 0.026]. No other significant differences were observed for residency, gender, chronic diseases, or economic status (Figs. 1 and 2).

**Fig. 3:**
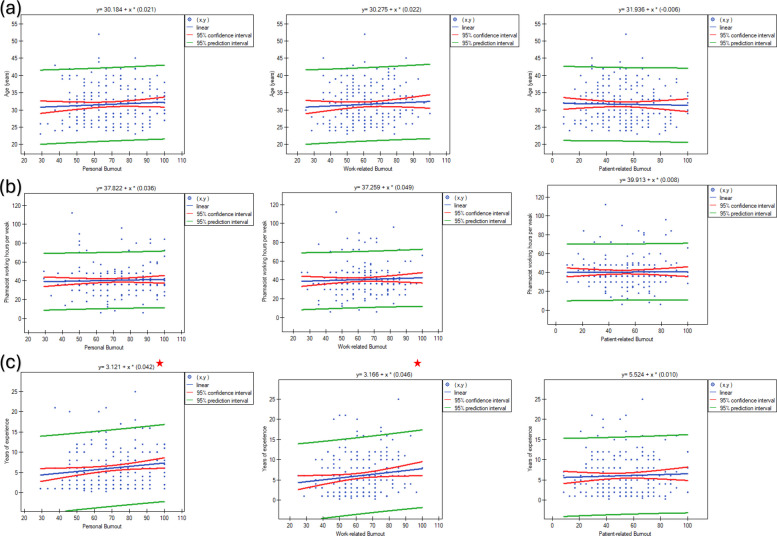
Spearman correlation to test differences in personal, work, and patient-related burnout among clinical pharmacists according to (**a**) age; (**b**) weekly working hours; and (**c**) years of experience. Red stars denote statistically significant differences

Spearman correlation analysis found that years of experience positively correlated with personal burnout (r = 0.180, *p* = 0.007) and work-related burnout (r = 0.144, *p* = 0.032). All other correlations were not statistically significant (Fig. 3).

These findings highlight a meaningful burden of burnout among Egyptian clinical pharmacists, with the majority experiencing moderate-to-high levels — a prevalence likely to impact well-being and job performance.

### Multivariate model of the three domains of burnout

#### Personal burnout

The multivariate model for personal burnout suggests that several factors have significant impacts. Years of experience (*p* = 0.025) positively correlates with burnout, indicating that more experienced individuals are likelier to experience burnout. Direct contact with patients (*p* = 0.021) also significantly increases burnout levels. Interestingly, being male (*p* = 0.040) is associated with lower burnout levels than being female. Lastly, individuals who have insufficient income and are in debt (*p* = 0.008) are significantly more likely to experience burnout than those who have enough income and can save. In this model, other factors such as age, working hours per week, having dependent children, residency, and having chronic diseases do not significantly predict burnout levels.

#### Work-related burnout

The multivariate model for work-related burnout suggests that years of experience (*p* = 0.042) and the highest education level (*p* = 0.025) are significant predictors. More years of experience are associated with higher levels of work-related burnout. Individuals with an M.Sc. degree compared to a B.Pharm are more likely to experience work-related burnout. Interestingly, individuals who have insufficient income and are in debt (*p* = 0.004) are significantly more likely to experience work-related burnout than those who have enough income and can save. Like the personal burnout model, other factors do not significantly impact work-related burnout.

#### Patient-related burnout

The multivariate model for patient-related burnout suggests that family income/economic level is a significant predictor. Specifically, individuals who have insufficient income and are in debt (*p* = 0.019) are significantly more likely to experience patient-related burnout than those who have enough income and can save. The other factors, consistent with the previous models, do not significantly influence patient-related burnout (3).

**Table 3:** Multivariate model for personal, work, and patient-related burnout

	**Model Coefficients -personal**				**Model Coefficients—work**				**Model Coefficients—patient-related**			
		**95% CI**				**95% CI**				**95% CI**		
**Predictor**	**Estimate**	**Lower**	**Upper**	**p**	**Estimate**	**Lower**	**Upper**	**p**	**Estimate**	**Lower**	**Upper**	**p**
Age (years)	−0.364	−1.032	0.303	0.283	−0.169	−0.744	0.406	0.564	−0.637	−1.444	0.170	0.121
Working hours per week	0.021	−0.134	0.176	0.789	0.033	−0.100	0.167	0.623	−0.036	−0.224	0.151	0.703
Years of experience	0.720	0.093	1.347	0.025	0.559	0.019	1.099	0.042	0.480	−0.278	1.237	0.213
Dependent children	−1.099	−3.442	1.243	0.356	−1.037	−3.055	0.981	0.312	0.974	−1.857	3.804	0.498
Having direct contact with patients												
Yes – No	9.026	1.348	16.704	0.021	4.242	−2.372	10.857	0.207	−2.200	−11.478	7.079	0.641
Gender												
Male – Female	−6.449	−12.598	−0.300	0.040	−1.589	−6.886	3.708	0.555	2.795	−4.637	10.226	0.459
Residency												
Urban – Rural	−2.188	−7.646	3.270	0.430	−2.357	−7.058	2.345	0.324	−4.302	−10.899	2.294	0.2
Family income/economic level												
Just enough – Enough and save	5.643	−1.026	12.312	0.097	1.992	−3.753	7.737	0.495	5.933	−2.126	13.992	0.148
Not enough – Enough and save	5.730	−1.535	12.995	0.121	4.899	−1.359	11.157	0.124	6.329	−2.450	15.109	0.157
Not enough and in debt – Enough and save	14.782	3.938	25.627	0.008	13.744	4.402	23.086	0.004	15.659	2.553	28.765	0.019
Highest education level												
M.Sc. – B.Pharm	3.545	−1.291	8.380	0.150	4.761	0.596	8.927	0.025	0.912	−4.931	6.756	0.759
Ph.D. – B.Pharm	−3.348	−13.06	6.364	0.498	−3.485	−11.85	4.881	0.412	−3.411	−15.147	8.325	0.567
Having chronic diseases												
Yes – No	0.365	−5.968	6.697	0.910	−3.948	−9.404	1.507	0.155	−0.258	−7.911	7.395	0.947

#### Predictors of having high burnout (overall score ≥ 75)

The univariate predictors for high overall burnout indicate that years of experience (*p* = 0.023) and financial status (*p* = 0.013 for those without enough income and debt) are significant factors. More years of experience and being in debt are associated with higher odds of burnout, with values of (OR = 1.08, 95% CI 1.01 to 1.15) and (OR = 6.00, 95% CI 1.46 to 24.63), respectively. Other factors, such as age, direct patient contact, gender, residency, income level (just enough or not enough), education level (M.Sc. or Ph.D.), working hours per week, and having dependent children, do not significantly predict high overall burnout in this model (Table 4).

**Table 4:** Univariate predictors and back stepwise multiple regression analysis results for having a high or above overall burnout score (≥ 75)

	**b coeff**	**b error**	**Wald stat**	**p-value**	**odds ratio**	−95% CI	** + 95%** **CI**
	Univariate predictors						
Age (years)	0.006	0.031	0.034	0.853	1.006	0.946	1.070
Are you in direct contact with the patient?	0.460	0.646	0.506	0.477	1.583	0.446	5.615
Gender	−0.827	0.559	2.192	0.139	0.437	0.146	1.307
Residency	−0.396	0.387	1.047	0.306	0.673	0.315	1.437
Family income/economic level[Just enough]	0.204	0.545	0.140	0.709	1.226	0.421	3.565
Family income/economic level[not enough]	0.385	0.581	0.439	0.507	1.469	0.471	4.585
Family income/economic level[not enough and in debt]	1.792	0.720	6.185	0.013	6.000	1.462	24.626
Highest education level [M.Sc.]	−0.213	0.353	0.364	0.546	0.808	0.405	1.614
Highest education level [Ph.D.]	−0.481	0.798	0.364	0.546	0.618	0.129	2.951
Pharmacist working hours per week	0.006	0.011	0.311	0.577	1.006	0.985	1.028
Years of experience	0.075	0.033	5.194	0.023	1.078	1.011	1.150
Dependent children	0.032	0.136	0.057	0.812	1.033	0.791	1.349
	Back stepwise regression model						
Years of experience	0.080	0.033	5.881	0.015	1.084	1.016	1.157

The back stepwise regression model for high burnout indicates that years of experience (*p* = 0.015) is a significant predictor. Increased years of experience are associated with higher odds of burnout, with an odds ratio of 1.08, suggesting that for each additional year of experience, the odds of high burnout increase by approximately 8.4% (Table 4).

## Discussion

### Summary of findings

This study identified a high prevalence of burnout among Egyptian hospital-based clinical pharmacists, with a substantial proportion meeting moderate to high burnout thresholds. Pharmacists with direct patient contact experienced significantly higher personal burnout, while those holding an M.Sc. degree reported higher work-related burnout than B.Pharm holders. Years of experience positively correlated with both personal and work-related burnout. Regression analysis showed that personal burnout was more prevalent among experienced individuals, those interacting directly with patients, females, and those in debt. Work-related burnout was associated with more years of experience, an M.Sc. degree, and debt, while patient-related burnout was predicted solely by debt. Overall, higher burnout was linked to greater professional experience and financial indebtedness.

### Burnout prevalence and severity

In our sample, 76% of pharmacists reported at least moderate overall burnout, and 21% met high or severe criteria. Personal burnout scores were highest, followed by work-related burnout, while patient-related burnout was comparatively lower. This pattern mirrors prior findings in clinical pharmacists, where emotional exhaustion consistently emerges as the most prominent dimension [7, 29]. Our prevalence rates are broadly consistent with U.S. data reporting a 61.2% burnout rate among hospital clinical pharmacists [7] and with rates exceeding 50% observed internationally [30]. However, they exceed those reported in some Middle Eastern contexts, such as Qatar, where burnout prevalence was comparatively lower [10]. Differences may reflect variation in workload intensity, role expectations, and healthcare system resources. The high burden observed here suggests a substantial risk to both workforce sustainability and patient care quality.

### Demographic factors and burnout levels

Male pharmacists reported lower personal burnout, consistent with previous studies showing higher stress and burnout in female pharmacists, potentially due to the dual demands of professional duties and family responsibilities [9, 31]. We found no association between age and burnout; the sample’s age range was narrow (median 30 years; IQR 27–35), which likely limited our ability to detect age effects**.** Residency location (urban vs. rural) also showed no differences, suggesting comparable working conditions across hospital settings.

Pharmacists with an M.Sc. degree exhibited higher burnout levels than those with a B.Pharm degree. Pharmacists with an M.Sc. degree often take on more complex roles and responsibilities, leading to a higher workload [32]. Moreover, the expectations and pressures of advanced professional roles may contribute to a poor work-life balance [29]. Continuous professional development, required for career advancement, can also add to the stress, particularly for those with M.Sc. degrees who are involved in research or teaching [30]. These factors, among others, have been previously reported to increase burnout [30]. This issue warrants further investigation, especially given that Egypt's pay for pharmacists with an M.Sc. degree in clinical pharmacy remains relatively low. Although the average salary for pharmacists in Egypt increases with higher education, the overall pay remains modest compared to international standards. Additionally, obtaining an M.Sc. degree in Egypt can be quite expensive, and the salary increase may not fully offset.

Direct patient contact was associated with significantly higher personal burnout. This is consistent with Kang et al. [31], who found that pharmacists spending more time in patient care reported greater stress, and with findings from community pharmacists where higher customer volumes were linked to burnout [9]. The emotional labour inherent in patient-facing roles – including managing expectations, addressing concerns, and maintaining empathy under pressure – likely contributes to this burden.

Financial status was a consistent predictor across all domains, with pharmacists in debt significantly more likely to report burnout. This aligns with existing knowledge; among clinical pharmacists, low salaries have been identified as a work-related stressor, and underappreciation is an independent risk factor for burnout [7]. This aligns with Egypt’s recent economic context: headline inflation peaked around 38% in Sept-2023 and, following the March-2024 exchange-rate float and sharp rate hike, the pound depreciated substantially before inflation gradually eased to about 12% by Aug-2025 – an arc that markedly compressed real wages [33]. Pharmacist remuneration in Egypt varies by sector and seniority; indicative sources suggest annual gross pay on the order of EGP 4k–21k/month (1 USD ≈ 48 EGP) for clinical pharmacists, with entry-level offers in some postings nearer EGP 4.5k–9k/month [34]. Meanwhile, both private and public sector minimum wages were raised to EGP 7,000/month in March–July 2025, underscoring policy efforts to buffer living-cost pressures [35]. In this environment, household debt/arrears likely amplify role stress – through extra shifts or moonlighting to service obligations, reduced recovery time, and perceived loss of control – mechanisms consistently linked to burnout. While our income measure captures subjective sufficiency rather than exact earnings, the combination of high costs and modest take-home pay provides a plausible pathway from financial hardship to burnout among clinical pharmacists in Egypt.

It is essential to highlight that work-related rewards can be diverse; however, a mismatch can heighten a sense of inefficacy and susceptibility to burnout. For instance, a salary that does not align with one's contributions and a lack of appreciation from patients, colleagues, or organizational leaders can undermine an individual's sense of accomplishment [36]. This underscores the need for financial wellness programs as part of comprehensive burnout prevention strategies.

Regarding the working hours, a systematic review by Dee et al. [30] found that more than half of pharmacists experience burnout and identified longer working hours, less professional experience, and high patient/prescription volumes as risk factors. In our sample, however, weekly working hours were not associated with any CBI domain (all p > 0.05), despite a mean of 40.3 ± 15.0 h (median 36; IQR 36–48). This null association may reflect (i) clustering around standard 36–48-h schedules, which reduces variability, and (ii) the limitation of weekly hours as a crude proxy for workload intensity (e.g., patient load, interruptions, night shifts/on-call). In public-sector hospitals, routine recruitment cycles may mitigate pharmacist understaffing and overtime, making long weeks uncommon in our cohort. By contrast, community pharmacies often operate 12-h shifts (≈60 h/week), which could contribute to the positive association between hours and burnout reported elsewhere. Future studies should model non-linear/threshold effects and incorporate objective workload metrics (patient volume per shift, night/on-call frequency) to characterize this relationship precisely.

### Predictors of having high burnout (CBI overall score ≥ 75)

During our analysis, we highlighted the years of experience as a critical factor because experienced pharmacists not only had higher personal and work-related burnout, but each additional year was associated with an ≈8% increase in the odds of high burnout (CBI ≥ 75; OR ≈ 1.08 per year). Several other factors may help explain this issue. Jones et al., in their study involving 974 clinical pharmacists [7], reported that the burnout rate was high and primarily driven by emotional exhaustion. They identified several subjective factors as predictors of burnout, including inadequate administrative and teaching time, uncertainty of health care reform, too many non-clinical duties, difficult pharmacist colleagues, and feeling that contributions are underappreciated. Similarly, the Durham et al. study involved 329 responses [29]; emotional exhaustion, depersonalization, and reduced personal accomplishment were among the risks of burnout. Another explanation was highlighted by Protano et al. [37]: the likelihood of burnout appears to be directly linked with job seniority. This is likely since senior pharmacists often bear greater responsibilities and experience increased fatigue, compounded by their prolonged years of patient interaction [37]. In the Egyptian context, we may also highlight that greater experience may not necessarily translate into improved financial stability or job satisfaction, which could help explain the positive association between tenure and burnout.

Our findings above contrast with Aljuffali et al. [9], who reported higher burnout among younger, less-experienced pharmacists. Several factors may explain the discrepancy. First, the two studies drew on different populations: our cohort comprised Egyptian hospital-based clinical pharmacists. Aljuffali et al. included a substantial proportion of community-pharmacy settings, where job demands and career trajectories differ. Second, “experience” was operationalised differently—our analysis used total years as a clinical pharmacist, while Aljuffali et al. grouped respondents into broader tenure categories that may have encompassed internship or pre-registration periods. Third, the studies were conducted at different points during the COVID-19 pandemic, which likely altered workload and staffing pressures in distinct ways. These differences suggest that the association between experience and burnout is context-dependent and should be interpreted cautiously.

## Strengths and limitations

This study has several strengths. To our knowledge, it is the first to assess burnout among clinical pharmacists in Egypt, addressing a clear gap in the regional literature. We achieved the target sample size and applied a validated instrument (CBI) to capture burnout across three distinct domains – personal, work-related, and patient-related – enabling a nuanced profile of risk. Multivariate modelling allowed us to identify independent predictors while adjusting for multiple covariates.

However, several limitations should be considered when interpreting our findings. First, the cross-sectional design precludes causal inference; all reported associations should be viewed as correlational. Second, the non-probability, snowball sampling approach may limit representativeness and is susceptible to self-selection bias, potentially over-representing pharmacists with strong views or greater online connectivity. Third, all data were self-reported, which introduces the possibility of recall bias and social desirability bias. Fourth, our sample did not cover all 27 governorates and was drawn predominantly from urban hospitals, restricting geographic generalisability and limiting intra-governorate comparisons. Fifth, although we included a range of demographic and work-context variables, some potentially important predictors – such as exact salary, seniority grade, subspecialisation, organisational culture, and staffing ratios – were not collected, leaving room for residual confounding. Finally, results from univariate and multivariate models should be interpreted carefully; certain predictors (e.g., gender) were statistically significant only in multivariate analysis, highlighting the importance of considering model specification and potential confounding effects. Taken together, these limitations suggest that the magnitude and direction of observed associations should be interpreted with caution.

## Conclusion

This first study of burnout among clinical pharmacists in Egypt reveals a substantial burden, with most respondents reporting at least moderate burnout and one in five meeting high or severe criteria. Independent predictors of higher burnout included financial debt, longer professional tenure, advanced academic qualifications, direct patient contact, and female gender. Debt and greater experience were the strongest predictors of high overall burnout.

Given the study’s cross-sectional, non-probability design, these findings should be interpreted as associations, not causation. Future research should employ probability sampling and longitudinal designs, incorporate objective workload and remuneration measures, and examine organisational and cultural determinants to clarify mechanisms and guide interventions.

In the meantime, healthcare facilities can mitigate burnout risk by co-designing targeted initiatives with senior pharmacists – such as workload redistribution, mentoring schemes, flexible scheduling, and financial wellness programs. Pharmacists reporting severe burnout should be promptly referred to occupational-health services or offered confidential support through the regional Pharmacists Syndicate.

## Data Availability

The datasets used and/or analyzed during the current study are available from the corresponding author upon reasonable request.

## Abbreviations

STROBE: Strengthening the Reporting of Observational Studies in Epidemiology
CBI: Copenhagen Burnout Inventory
OR: Odds ratio
CI: Confidence interval
SD: Standard deviation
IQR: Interquartile range
